# Sciatic Nerve Injury Secondary to a Gluteal Compartment Syndrome

**DOI:** 10.7759/cureus.9012

**Published:** 2020-07-05

**Authors:** Adel Hanandeh, Ahmed A Shamia, Max Murray Ramcharan

**Affiliations:** 1 General Surgery, Columbia University College of Physicians and Surgeons at Harlem Hospital Center, New York, USA; 2 Surgery, Harlem Hospital Center, New York, USA

**Keywords:** gluteal pain, thigh pain, gluteal swelling, gluteal compartment syndrome, gluteal fasciotomy, ivda, prolong immobilization, compartment syndrome, sciatic neuropathy, fasciotomy

## Abstract

Gluteal compartment syndrome (GCS) is extremely rare, with a low index of suspicion among physicians, hence, it is highly overlooked. The underdiagnosis can lead to irreversible tissue ischemia and severe neurological deficits. GCS is a surgical emergency and requires immediate surgical intervention given its high morbidity and mortality. Based on the limited available literature, multiple etiologies have been postulated including traumatic and nontraumatic causes. This article presents a complex and unusual case of GCS after prolonged immobilization in an IV drug abuser who was subjected to initial missed diagnosis.

## Introduction

Compartment syndrome (CS) can be defined as an elevated pressure in a closed space that compromises the tissue viability secondary to ischemia. The overall incidence of CS in the current literature is 7.3 per 100,000 in males and 0.7 per 100,000 in females. The most common spaces involved are lower extremity followed by abdomen, upper extremity, and rarely the gluteal region. While there has been extensive published literature about the CS of the extremity and abdomen rendering adequate resources to the physicians for its optimal management, there has been limited available literature on the CS of the gluteal region, This in turn translates to decreased awareness and knowledge among physicians, resulting in a low index of suspicion and missed diagnosis compromising patient care [1-3].

Gluteal compartment syndrome (GCS) is an uncommon cause of gluteal and thigh pain and often unrecognized. There are multiple causes of GCS including traumatic and nontraumatic causes described in the literature. The incidence of GCS has been the highest among middle-age men especially drug abusers likely secondary to prolonged immobilization. GCS etiologies include anticoagulation, obesity, bone marrow biopsy, pelvic trauma, posterior hip dislocation, and incorrect position during surgeries with long operative time and epidural anesthesia [4]. 

The GCS clinical manifestations are similar to those of other CSs such as pain of proportion to the physical exam, paresthesia, sciatic nerve palsy, massive rhabdomyolysis, multiple organ dysfunction, including kidney failure, and finally tense compartments. GCS diagnosis is often clinical, but measurement of gluteal compartment pressure may be helpful in unresponsive patients where the degree of symptoms such as pain or paresthesias cannot be assessed. Radiographical studies, including ultrasound, CT, and MRI are usually omitted in order to prevent any delay in treatment. The gold standard for treatment is emergent fasciotomy [4-6].

## Case presentation

This is a 38-year-old polysubstance abuser male who presented to the ED with a complaint of sudden onset of right lower extremity weakness upon waking up. The patient admitted to being under the influence of heroin, cocaine, and alcohol. The patient did not endorse any history of trauma but described sleeping on the floor for many hours prior to presentation. On exam the patient was hemodynamically stable, alert, and oriented to time, place, and person. Pertinent physical exam findings include right periorbital swelling, left lower chest tenderness, right thigh tenderness, swelling, and redness without any signs of external trauma. Notably the patient was found to have significant right lower extremity weakness, and diminished sensation.

**Figure 1:**
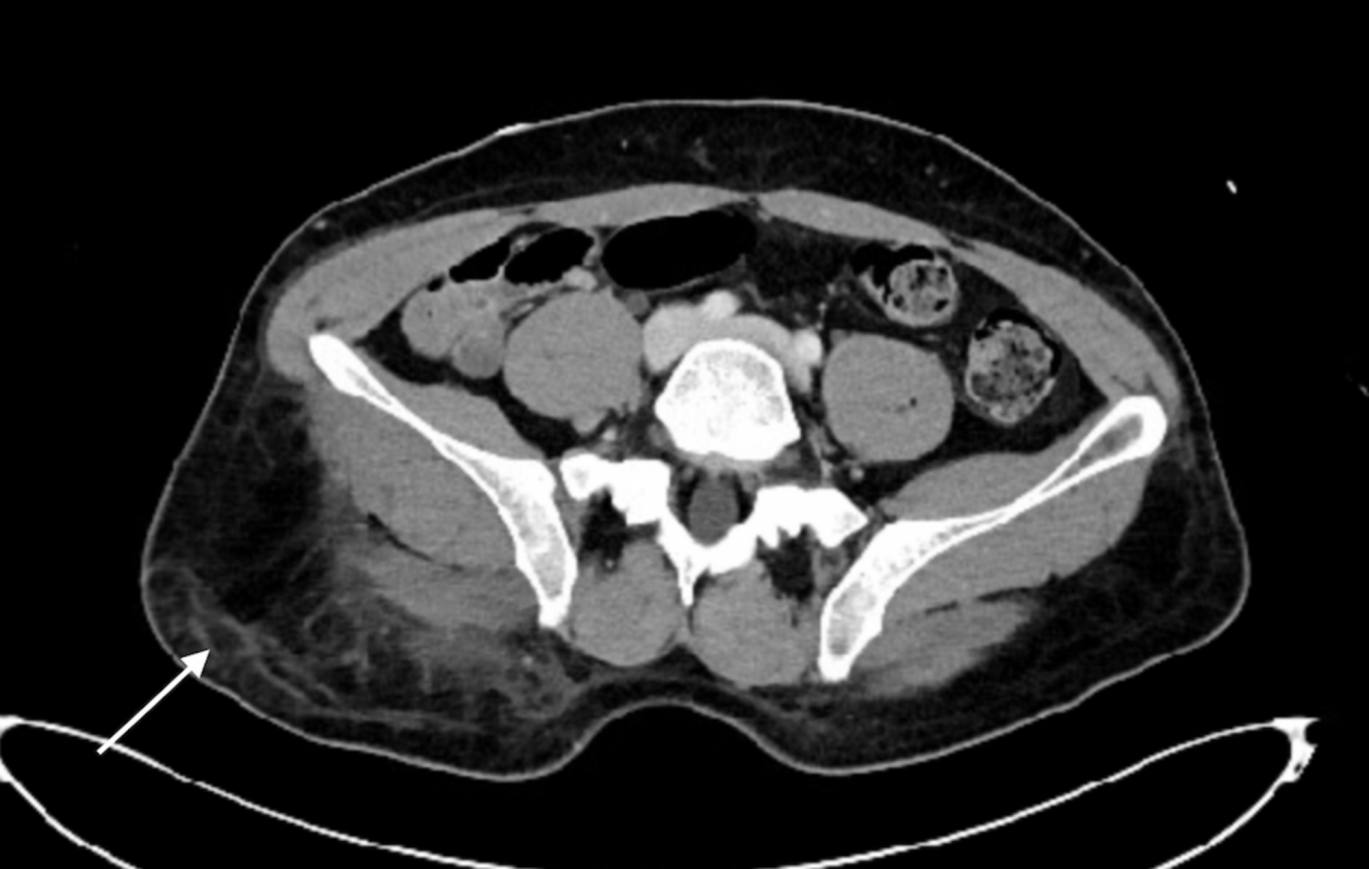
Axial view CT abdomen and pelvis illustrating the extent of right gluteal edema and soft tissue infiltrate.

**Figure 2:**
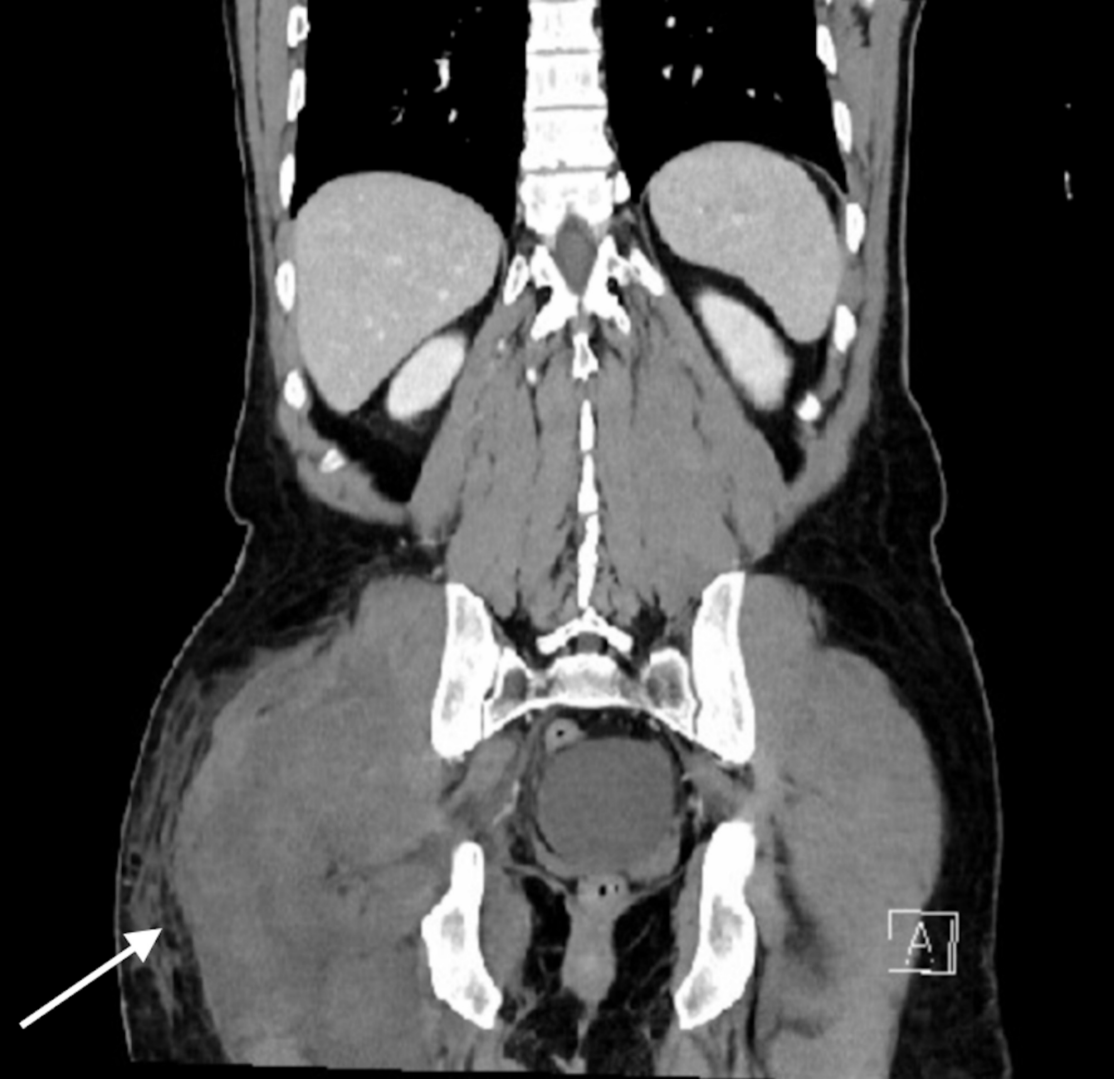
Coronal view of CT abdomen and pelvis illustrating a right gluteal soft tissue edema and infiltrate.

Laboratory values indicated normal basic metabolic panel, borderline elevated white blood cell (WBC), deranged liver function tests (LFTs), and a creatine phosphokinase (CPK) of 44,543. The drug screen was positive for methadone, tetrahydrocannabinol (THC), and alcohol. A CT scan of the abdomen and pelvis was obtained which indicated right gluteal soft tissue swelling with subcutaneous fatty infiltration. At that time the patient was admitted to the floor for conservative management and hydration (Figures 1-2).

Twenty-four hours later, the patient became agitated and was complaining of severe right buttock pain. Upon further exam, the patient was found to have a tense right buttock with erythema, edema, and pain out of proportion to physical exam. Right lower extremity at that time was completely paralyzed. At this point the surgery team was convinced that the patient had developed a gluteal/thigh CS. Hence, emergently the patient was taken to the operating room for a right thigh and gluteal fasciotomy. 

**Figure 3:**
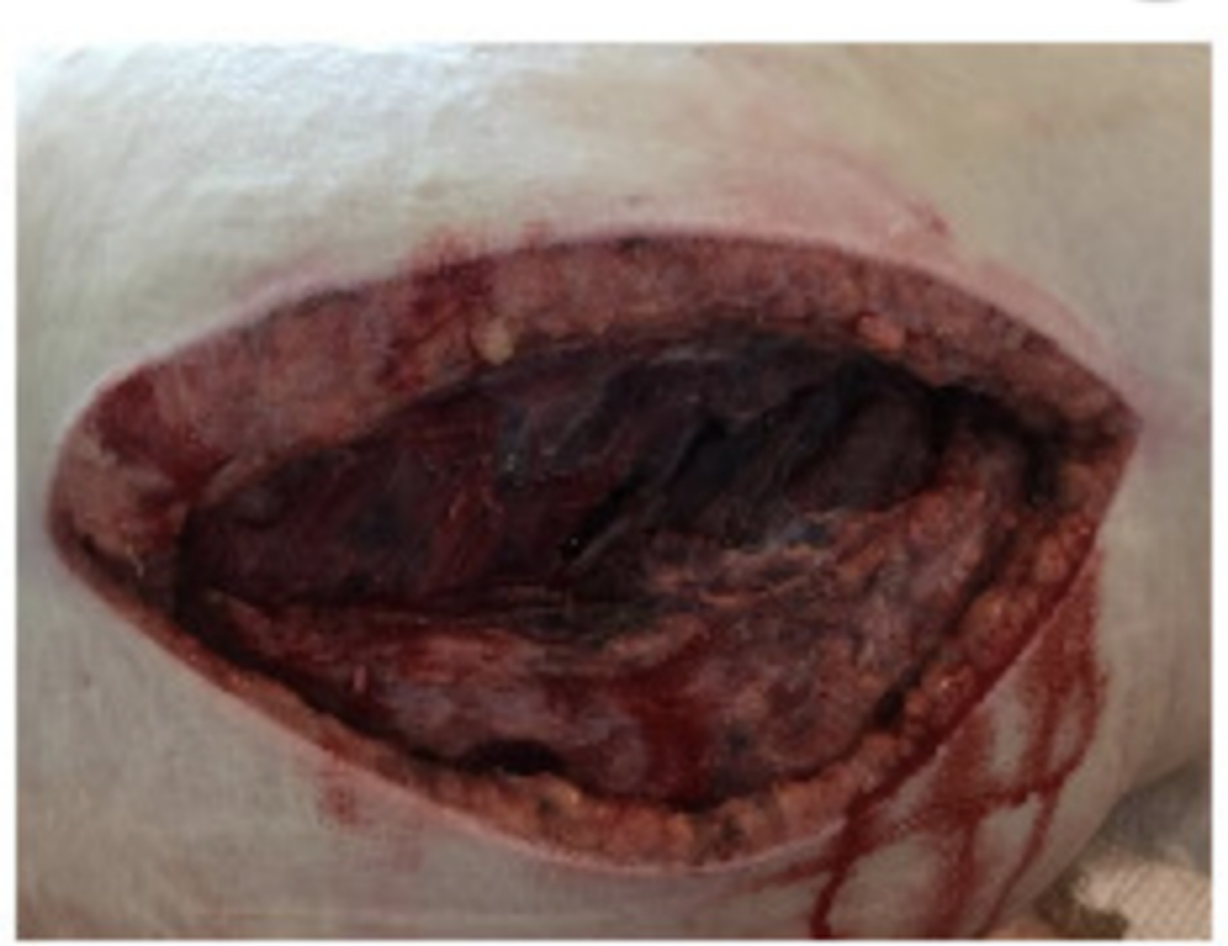
Image of the surgical incision extending from the upper one third of the thigh and curved to posterior medial aspect of the buttock.

In the operating room the patient was placed in left lateral decubitus position and a 10 cm incision on the right lateral thigh was entertained. An incision was made 8 cm inferior to the anterior superior iliac spine in the middle of the lateral aspect of the thigh and extended to the proximal upper thigh until tensor fasciae latae fibers were encountered and vertically released. A small hematoma was evacuated. Yet, the right buttock remained swollen and tense; therefore, the incision was extended superiorly by 5 cm at 150° angle toward the posterior-medial aspect of the buttock resembling Kocher-Langenbeck approach; in which a subcutaneous flap was made on the posterior medial edge of the wound allowing for better access to the gluteus muscle compartments. Upon achieving a better view, the superficial, deep, and intermuscular fascia of the gluteus was divided allowing for further evacuation of deep gluteal hematoma and release of the tension. Of note, this technique had not been previously described in the literature for the gluteal compartment release but was carried in this case due to the presence of combined thigh CS and GCS (Figure 3).

The patient tolerated the procedure well and was transferred to the floor. Plastic surgery was consulted for wound closure and a wound vac was placed. Few weeks later the patient reported much improvement in his pain and his neuropraxia. Yet, he developed permanent nerve deficit and weakness in the affected extremity. 

## Discussion

Compartment syndrome is a rare type of compartment which is usually underdiagnosed and can lead to irreversible tissue ischemia and neurological deficits. GCS is a surgical emergency and requires immediate surgical intervention given its high morbidity and mortality. GCS pathophysiology is similar to that of other CSs that are caused by an increase in the interstitial pressure in a nonexpandable musculoskeletal compartment, which can occur due to the direct result of fluid accumulation within that confined space or due to any external restrain which may prohibit the expansion of the compartment. Ultimately, it leads to increase in the internal pressure of the compartment, with subsequent malperfusion and tissue ischemia [1-3].

A compartment pressure capable of compromising perfusion is usually within 10-30 mmHg of diastolic pressure; at such a pressure the muscle oxygenation decreases, and tissue injury may develop. Given that the nervous system is the most sensitive to tissue hypoxia, it has been described that a nerve conduction disturbance emerges when the difference between the compartment and diastolic pressure becomes less than 30 mmHg or compartment pressure becomes greater than 30 mmHg [3]. A further rise in the compartment pressure may ultimately lead to a compromise in the circulation leading to frank tissue necrosis. Hence, a vicious cycle ensues by restricting local tissue perfusion by reducing the arteriovenous pressure gradient. The degree of tissue damage depends on the duration of extremity ischemia and the metabolic rate of the tissue, but generally, irreversible damage ensues after four to eight hours [4-6]. 

Multiple studies in the literature described the estimated ischemia time that would result in irreversible neurological injuries. Whitesides study reported that four-hour ischemic time can lead to irreversible muscle damage. Given that the nervous system is generally more sensitive to hypoxia, it has been described that a 33-minute nervous tissue hypoxia time can result in irreversible injuries. Other studies have reported that eight hours of muscle ischemia cause irreversible damage. Given the seriousness of this syndrome, immediate identification and surgical intervention are required to decrease morbidity [6-8].

Given the seriousness of this syndrome, it is crucial to identify the early and late manifestation of the GCS. Usually, symptoms progress through paresthesias, anesthesia, frank paralysis, and finally diminished pulses and ischemic changes. Similar to that of any CS, GCS can have multiple systemic sequelae such as severe rhabdomyolysis characterized by myoglobinemia, myoglobinuria, acute kidney injury with elevated blood urea nitrogen (BUN), and creatinine. Hence to prevent such sequelae aggressive fluid resuscitation and urine alkalinization can be utilized [9-12].

In the current literature, GCS has been attributed to a variety of etiologies including traumatic such as pelvic blunt and penetrating injuries, posterior hip dislocation, injection and bone marrow biopsies. Other etiologies included medication induced (such as anticoagulants and statins), infectious, coagulopathies, sickle cell disease, and finally connective tissue disorders (such as Ehlers-Danlos syndrome). The most common etiology of GCS is related to prolonged local pressure on the gluteal muscle’s secondary to immobilization. The Henson systematic review included multiple etiologies that contributed to GCS, of which prolonged immobilization (in IVDA) was the number one cause (50%), followed by posttotal joint arthroplasty with epidural anesthesia (21%), trauma (21%), and necrotizing fasciitis (7%) [9-12].

The anatomy of a gluteal region involves three compartments from lateral to most medial including the tensor fasciae latae, gluteus medius and minimus, and finally gluteus maximus. It is crucial to release these three compartments to provide adequate decompression of the area [4].

Tensor fasciae latae muscle originates from the anterior superior iliac spine and the anterior part of iliac crest. Along with the gluteus maximus it forms the Iliotibial tract, which attaches to the lateral condyle of the tibia. It is a long muscle with a length of 15 cm overlying the gluteus minimus and medius and is supplied by the superior gluteal artery and nerve. Gluteus medius and minimus muscles arise from the ilium and extend to the greater trochanter and are supplied by the superior gluteal artery and nerve. While the gluteus maximus is the most superficial muscle in the buttock and is innervated by the inferior gluteal nerve and the superior and inferior artery, the muscle extends from proximal ilium to the proximal posterior femur along the iliotibial tract [5-8].

Neurological manifestation of GCS usually arises due to sciatic nerve injury. Due to its anatomical location (below the piriformis and over the obturator internus muscle), it is highly susceptible to compression by swelling of the gluteal muscles resulting in an early manifestation of neuropraxia. In many cases of GCS release fasciotomies surgeons elect to perform neurolysis of the sciatic nerve to prevent any tension secondary to the postfasciotomy inflammatory adhesions [5, 8-12].

Gluteal compartment syndrome similar to other CSs is an outcome of fluid accumulation likely of blood illustrating a confined hematoma. Such a gluteal hematoma usually results from vascular injury to superior gluteal artery, medial circumflex femoral arteries and to a lesser extent the inferior gluteal artery. [9]

Finally, the ultimate therapy for CS is a surgical fasciotomy. Given the scarce number of cases reported, there is no defined standard surgical fasciotomy procedure. Many surgeons electively perform longitudinal incisions along the lateral thigh and buttock area, allowing access to all the compartments. Many case reports have described the use of Kocher-Langenbeck approach or the modified Gibson approach [3-6].

The Kocher-Langenbeck approach is accomplished through a skin incision starting few centimeters lateral to the posterior superior iliac spine extending distally and anteriorly over the greater trochanter towards the lateral aspect of the femoral shaft. The incision usually extended distal to the insertion of the gluteus maximus tendon. The modified Gibson is a straight skin incision, in the plane between the anterior border of the gluteus maximus muscle and the tensor fasciae latae in which a long straight lateral incision is made 10-15 cm proximal to the trochanter and extends 10-15 cm along the proximal lateral femur. Thus, the tensor fasciae latae fibers are divided longitudinally in both the approaches and the superficial gluteus maximus is reflected posteriorly to reveal the underlying deep anatomic structures. The advantages of the modified Gibson posterior approach over the Kocher-Langenbeck include increased anterosuperior direct access and decreased risk of iatrogenic injury to the nerve supply to the gluteus maximus muscle [6-8].

In our case, the GCS was initially missed due to the lack of concern for a CS in that region which led to a compromise in patient care resulting in right lower extremity neuropraxia. It is important to note that the CT scan finding was not conclusive and appeared as if the patient had a soft tissue infection. 

Our surgical approach was initially inadequate until we realized that all compartments needed to be released. Hence it better to always plan to release all compartments instead of taking a more conservative approach, as a conservative approach increases the risk of not releasing one of the congested muscular compartments. 

## Conclusions

Gluteal compartment syndrome is an extremely rare condition that can be easily overlooked, especially in unconscious patients. In an awake and responsive patient, swelling and tautness should mandate further investigation and a CS should be high on the list of differentials.

Trauma surgeons must immediately recognize the possibility of GCS in patients who present with severe gluteal pain especially when presenting after acute pelvic trauma or prolonged immobilization. Any delay in diagnosis or treatment can be devastating, leading to permanent disabilities including, irreversible loss of gluteal muscles, sciatic nerve palsy, or even end-stage kidney failure. Thus, this case highlights the importance of early diagnosis and treatment of this uncommon condition.
